# Fabric soft pneumatic actuators with programmable turing pattern textures

**DOI:** 10.1038/s41598-024-69450-z

**Published:** 2024-08-19

**Authors:** Masato Tanaka, Yuyang Song, Tsuyoshi Nomura

**Affiliations:** 1 Toyota Central R&D Laboratories, Inc., 41-1, Yokomichi, Nagakute, Aichi 480-1192 Japan; 2 Toyota Research Institute of North America, Toyota Motor North America, Ann Arbor, MI 48105 USA

**Keywords:** Mechanical engineering, Software, Computer science

## Abstract

This paper presents a novel computational design and fabrication method for fabric-based soft pneumatic actuators (FSPAs) that use Turing patterns, inspired by Alan Turing’s morphogenesis theory. These inflatable structures can adapt their shapes with simple pressure changes and are applicable in areas like soft robotics, airbags, and temporary shelters. Traditionally, the design of such structures relies on isotropic materials and the designer’s expertise, often requiring a trial-and-error approach. The present study introduces a method to automate this process using advanced numerical optimization to design and manufacture fabric-based inflatable structures with programmable shape-morphing capabilities. Initially, an optimized distribution of the material orientation field on the surface membrane is achieved through gradient-based orientation optimization. This involves a comprehensive physical deployment simulation using the nonlinear shell finite element method, which is integrated into the inner loop of the optimization algorithm. This continuous adjustment of material orientations enhances the design objectives. These material orientation fields are transformed into discretized texture patterns that replicate the same anisotropic deformations. Anisotropic reaction-diffusion equations, using diffusion coefficients determined by local orientations from the optimization step, are then utilized to create space-filling Turing pattern textures. Furthermore, the fabrication methods of these optimized Turing pattern textures are explored using fabrics through heat bonding and embroidery. The performance of the fabricated FSPAs is evaluated through three different deformation shapes: C-shaped bending, S-shaped bending, and twisting.

## Introduction

Fabric-based soft pneumatic actuators (FSPAs) can easily alter their shapes under simple positive or negative pressure and produce pre-programmable motion^1^. The intrinsic flexibility of these soft robotic systems provides distinct advantages over traditional rigid robotic architectures. They are economically more feasible, lightweight, demonstrate superior compliance, and maintain enhanced safety for interactions within uncertain environments and in proximity to the human physique^2,3^. Consequently, these soft robotic technologies are increasingly recognized for a wide array of potential applications. This includes soft manipulators^4^, advanced airbags^5,6^, mylar balloons^7,8^, emergency shelters^8^, the emulation of biological movement in robotics^2,9^, navigational adaptability in unstructured environments^9,10^, the nuanced handling and manipulation of diverse objects^11^, and devices designed for therapeutic and assistive purposes^3,12^.

Despite the appealing properties of FSPAs, there has not been sufficient exploration into designs with more complex behaviors, and only simple geometries have been considered. In the context of designing motions for soft robotics, previous efforts have primarily focused on embedding and combining the physical properties and responsiveness of materials such as hydrogels^13–16^, liquid crystal elastomers^17^, pre-stretched polymer filaments^18^, and inflatable structures^19–21^ with origami and kirigami approaches^22–24^. However, these efforts have not fully exploited the potential of programmable surface geometries.

The primary motivation for this study arises from the identified need for actuators in soft robotics capable of performing more complex, controlled movements without the requirement for special materials and technologies, but solely through the design of surface texture patterns on their membranes. This results in simple and low-cost implementations of FSPAs, as only two materials, hard and soft fabrics, are used. The current offerings of textile-based actuators in the market lack the capability to perform such dynamic functions, primarily due to limitations in design and fabrication techniques that do not allow for precise programming of material behavior at the micro-level.

In this context, Turing patterns could be one possible solution for designing surface texture patterns that induce more complex, controlled movements. The current study combines orientation optimization techniques with a de-homogenization method inspired by the Turing pattern. This approach not only efficiently addresses the technical challenges but also closely mimics intricate patterns found in nature. The Turing patterns, derived from Alan Turing’s seminal work^25^ on morphogenesis, describe naturally occurring designs that can be observed across various spatial scales in nature-from the skin of lizards^26^ and fish skins^27,28^ to the ridges of sand dunes^29^. While these natural designs are being emulated and exploited as aesthetic applications in industries such as fashion, the proposed method offers enhanced functionality and adaptability in the application of FSPAs.

Recently, Maestre et al.^30^ approached the development of robotic skins, specifically the ’ToRos’ project, through a method of topology optimization using a three-field filtering approach. While their work contributed significantly to the field, the current study introduces an entirely different approach to programming and manufacturing FSPAs. The proposed method begins with an optimization of material orientation distribution on the surface membrane, achieved through gradient-based orientation optimization. This involves a comprehensive physical deployment simulation using the nonlinear shell finite element method, integrated directly into the optimization algorithm. The goal is to continually adjust material orientations to meet specific design objectives. Following this, the material orientation field is converted into a texture pattern. This pattern replicates the same anisotropic deformation in the membrane, achieved by employing anisotropic reaction-diffusion equations to generate space-filling Turing pattern textures. In contrast to the traditional trial-and-error approach to achieve the desired shape when inflated^31^, the automatic design of these structures is realized using advanced numerical optimization techniques.

Recently, Tanaka et al.^32^ introduced a similar design approach, combined with fabrication using grayscale Digital Light Processing (g-DLP) 3D printing technology. However, this fabrication method is limited by the size of prototypes that can be produced with current DLP 3D printing techniques^33,34^. Consequently, this paper explores two alternative fabrication methods utilizing fabrics. The first method involves cutting optimized Turing pattern shapes from stiff fabric (Dyneema film) with a laser cutter and attaching them to a soft fabric (Thermoplastic Polyurethane, TPU)-based soft pneumatic actuator as reinforcement patches. These patches are then bonded with the base fabric by heat press machine. The second method utilizes embroidery for fabrication. In this approach, areas with optimized Turing pattern shapes are programmed using digitizing software specifically designed for embroidery. These designated areas are then stitched onto soft fabric (Polyurethane polymer) using a stiff thread (Kevlar thread, K-tech 75 Tex, 3 layers), applied by a needle.

By integrating Turing patterns with a novel fabrication technique that utilizes heat bonding or embroidery, this study not only bridges the research gap but also proposes a scalable method for producing FSPAs that can be customized for a broad range of applications, from wearable technology to deployable emergency shelters. This method represents a transformative step forward in the design and production of soft actuators, setting a new standard for what can be achieved in soft robotics.

## Theory

### Design method of FSPAs using orientation optimization

In proposed method, the distribution of material orientations is first optimized using a gradient-based optimization technique integrated with nonlinear shell finite elements to transform the structures into desired shapes during inflation. An objective function is defined by minimizing the Euclidean distance between the target and deformed shapes. The optimizer then iteratively modifies the distribution of material orientation guided by the gradients.

In the orientation optimization, a transversely isotropic material model is employed to represent the anisotropic physical properties characterized by a single preferred direction. The following six components ($a_{11},a_{22},a_{33},a_{12},a_{23},a_{13}$) of a symmetric orientation tensor $\textbf{a}$ are considered as design variables.

1 $$\begin{aligned} \begin{aligned} \begin{bmatrix} \textbf{a} \end{bmatrix} = \begin{bmatrix} a_{11}&{}a_{12}&{}a_{13}\\ a_{12}&{}a_{22} &{}a_{23}\\ a_{13}&{}a_{23}&{}a_{33} \\ \end{bmatrix}. \end{aligned} \end{aligned}$$

These components are not independent and are subject to the first, second, and third tensor invariant conditions, denoted as $I_1, I_2$, and $I_3$ respectively, as follows:

2 $$\begin{aligned}{} & {} I_1 = {{\,\textrm{tr}\,}}(\textbf{a}) = a_{11} + a_{22} + a_{33} = 1, \end{aligned}$$

3 $$\begin{aligned}{} & {} I_2=\begin{vmatrix} a_{22}&a_{23} \\ a_{23}&a_{33} \end{vmatrix} + \begin{vmatrix} a_{11}&a_{12} \\ a_{12}&a_{22} \end{vmatrix} + \begin{vmatrix} a_{11}&a_{13} \\ a_{13}&a_{33} \end{vmatrix} =0, \end{aligned}$$

and

4 $$\begin{aligned} I_3=\det (\textbf{a}) =0. \end{aligned}$$

In this study, the following constraint is used:

5 $$\begin{aligned} a_{ij}^2&=a_{ii}a_{jj}\quad \text{ for } (i,j)=\left\{ (1,2),(1,3),(2,3)\right\} , \end{aligned}$$

which satisfies the second and third invariant constraints (3) and (4).

Here, the fourth-order elastic tensor of the transversely isotropic material, denoted as $\textbf{C}^t$, is presented in the Voigt notation as follows:

6 $$\begin{aligned}{}[\textbf{C}^t]=\begin{bmatrix} C^t_{1111}&{}C^t_{1122}&{}C^t_{1122}&{}0&{}0&{}0\\ &{}C^t_{2222}&{}C^t_{2233}&{}0&{}0&{}0\\ &{} &{}C^t_{2222}&{}0&{}0&{}0\\ &{} &{} &{}C^t_{2323}&{}0&{}0\\ &{} \text{ symm. } &{} &{} &{}C^t_{1212}&{}0\\ &{} &{} &{} &{} &{}C^t_{1212} \end{bmatrix}. \end{aligned}$$

The elastic tensor $\textbf{C}^t$ is rotated by the orientation tensor $\textbf{a}$ in the following manner:

7 $$\begin{aligned} C^r_{ijkl}&= {} B_1 (\textbf{a})_{ij}(\textbf{a})_{kl} +B_2 \left[ (\textbf{a})_{ij}\delta _{kl} + (\textbf{a})_{kl}\delta _{ij}\right] + B_3\left[ (\textbf{a})_{ik}\delta _{jl} + (\textbf{a})_{il}\delta _{jk} + (\textbf{a})_{jk}\delta _{il} + (\textbf{a})_{jl}\delta _{ik}\right] \nonumber \\ & \quad + B_4(\delta _{ij}\delta _{kl}) + B_5(\delta _{ik}\delta _{jl}+\delta _{il}\delta _{jk}), \end{aligned}$$

where $\delta _{ij}$ represents Kronecker’s delta, and the coefficients $B_i$ are determined by $C^t_{ijkl}$, which are defined using the Young’s moduli of both the stiffer and softer materials; see the detailed equations in the previous work^32^, Section S2 of the Supplementary Materials. The Poisson’s ratios for the stiffer and softer materials are assumed to be 0.49 ($\nu _f=0.49$, $\nu _m=0.49$). Additionally, the volume fractions for both the stiffer and softer materials are $V_f=0.5$ and $V_m=0.5$, respectively, for stiffer and softer materials.

### Algorithm of orientation optimization

The material orientations are designed element-wise in Finite Element Method (FEM), and the overall optimization can be summarized as follows:

8a $$\begin{aligned} \displaystyle \mathop {\hbox {minimize}}_{a_{ij}(\textbf{x})}\qquad&\displaystyle J(\textbf{u}) \end{aligned}$$

8b $$\begin{aligned} \displaystyle \hbox {subject to}\qquad& \displaystyle a_{ij}(\textbf{x})\in [\delta _{ij}-1,1] \end{aligned}$$

8c $$\begin{aligned}& \displaystyle g_{1}:=a_{11}+a_{22}+a_{33}- 1\leq 0 \end{aligned}$$

8d $$\begin{aligned}&\displaystyle g_{2}:= a_{ij}^2 - {a_{ii}a_{jj}} = 0 \text{ for } (i,j)=\left\{ (1,2),(1,3),(2,3)\right\} \end{aligned}$$

8e $$\begin{aligned}&\text {Governing equation of inflation of shell structure} \end{aligned}$$

8f $$\begin{aligned}&\text { Rotated transversely isotropic tensor (7)}, \end{aligned}$$

where *J* represents the objective function, $\textbf{x}$ is the position vector within a fixed design domain, and $\textbf{u}$ denotes the displacement fields. These fields are obtained by solving the nonlinear shell static equilibrium equations, wherein the material properties are coupled with the orientation tensor $\textbf{a}$. To circumvent numerical instability during optimization, which often occurs in angular or vector representations due to the cyclic nature of trigonometric functions, the constraint on the first tensor invariant is modified as described in equation (8c). Specifically, the sum of the diagonal components of the orientation tensor $\textbf{a}$ is allowed to be less than one, denoted as $g_1 \le 1$. Further technical details concerning orientation optimization are extensively discussed in the references^35,36^.

For computing the deployed surface upon inflation, the inflation of thin-shell structures is simulated using geometrically nonlinear FEM.

The Total Lagrangian formulation is utilized to accommodate large displacements and finite rotations. Internal pressure is modeled as a follower force, consistently acting in the normal direction of the surface and maintaining a uniform magnitude, as detailed in the reference^37^.

The incremental-iterative technique using the full Newton–Raphson method is used to solve the nonlinear finite element program. The design variables $a_{ij}$ are given at the nodes in the FE analysis. The objective function *J* is the Euclidean norm of displacement at specific node points, as explained in Results section. The optimization problem Eqs. (8) is solved with gradient-based MMA (Method of Moving Asymptotes^38^), which is a standard mathematical programming method for structural optimization. This gradient-based MMA optimizer is integrated with sensitivity analysis of nonlinear shell finite elements designed to optimize the distribution of material orientations on the surface membrane. This approach is utilized to transform the structures into desired shapes during inflation by continuously adjusting material orientations to meet specific design objectives.

### Generation of turing pattern

In general, directly 3D printing anisotropic materials, which allow for control over local material orientation, is a complex task. Special post-processing may be necessary to utilize such 3D printing techniques based on the optimized distribution of material orientation, while also ensuring continuity.

In this study, Turing pattern textures are employed to print anisotropic material fields on the surfaces of inflatable structures. these material orientation fields are transformed into discretized texture patterns that can induce similar anisotropic deformations. The binarized Turing pattern textures are generated using solutions from anisotropic reaction-diffusion equations^39^. These equations involve two variables, *U* and *V*, representing two interacting hypothetical chemical substances. In this context, *U* and *V* correspond to stiff and soft material parts, respectively, with each having a volume fraction of 0.5. The anisotropic reaction-diffusion equations are given by

9 $$\begin{aligned} \frac{\partial U}{\partial t}= & {} \nabla \cdot \left( \textbf{D}_u \nabla U\right) + R_u(U,V), \nonumber \\ \frac{\partial V}{\partial t}= & {} \nabla \cdot \left( \textbf{D}_v \nabla V \right) + R_v(U,V), \end{aligned}$$

where $\partial /\partial t$ represents the material derivative, $\nabla$ denotes the gradient operator, while $R_u(U,V)$ and $R_v(U,V)$ are interactive reaction terms. Additionally, $\textbf{D}_u$ and $\textbf{D}_v$ are anisotropic diffusion coefficients.

The reaction terms $R_u(U,V)$ and $R_v(U,V)$ are augmented as

10 $$\begin{aligned} R_u(U,V)= & {} a_u U+ b_u V + c_u -d_uU, \nonumber \\ R_v(U,V)= & {} a_v U+ b_v V + c_v-d_vV, \end{aligned}$$

where $a_u$, $b_u$, $c_u$, $d_u$, $a_v$, $b_v$, $c_v$ and $d_v$ are constant parameters. The diffusion coefficients $\textbf{D}_u$ and $\textbf{D}_v$ are expressed in terms of the normalized fluid flow velocity vector $\bar{\textbf{u}}$ as

11 $$\begin{aligned} \textbf{D}_u= & {} (L_u-W_u)\bar{\textbf{u}}\otimes \bar{\textbf{u}}+W_u \textbf{I}, \nonumber \\ \textbf{D}_v= & {} (L_v-W_v)\bar{\textbf{u}}\otimes \bar{\textbf{u}}+W_v \textbf{I}, \end{aligned}$$

where $\textbf{I}$ is second-order identity tensor, $\otimes$ represents dyadic product operator, and $L_u, W_u, L_v, W_v$ are given by

12 $$\begin{aligned} L_u = l_u^2 W_u, \quad W_u=(w_uw)^2, \quad L_v = l_v^2 W_v, \quad W_v=(w_vw)^2. \end{aligned}$$

The magnitudes of the anisotropy parameters for *U* and *V* are denoted by $l_u$ and $l_v$, respectively. Similarly, $w_u$ and $w_v$ represent the channel pitch parameters for *U* and *V*, while *w* indicates the lateral magnitude of diffusion. These parameters influence the resulting space-filling Turing pattern. In this study, the following values were used: $l_u=1$, $l_v=1$, $w_u^2=0.02$, $w_v^2=0.5$, $w=0.12$, $a_u=0.08$, $b_u=0.08$, $c_u=0.04$, $d_u=0.03$, $a_v=0.1$, $b_v=0$, $c_v=-0.15$ and $d_v=0.08$. In Equation (11), the term $\bar{\textbf{u}}\otimes \bar{\textbf{u}}$ is substituted with the orientation tensor $\textbf{a}(\textbf{x})$, which is derived during the orientation optimization step. The anisotropic reaction-diffusion equations, as defined in (9), are computed as a function of time *t* until an equilibrium state is achieved for the reaction-diffusion of the chemical substances *U* and *V*. This process ultimately results in the formation of the space-filling binarized Turing pattern.

## Methods

Based on optimization design guidelines, fabrication methods are investigated to realize the optimized Turing-patterned FSPAs. First, a fabrication methods using heat press and bonding are evaluated. The stiffness difference between the Turing pattern material and the base material should be as large as possible to achieve significant actuation motion. It is challenging to find two fabric materials that have a substantial contrast in stiffness and can be heat-pressed together. Dyneema is currently the fabric with the highest modulus available, while TPU is used to maintain bonding strength and airtightness. Embroidery is another alternative fabrication method, where variable materials can be sourced to meet the design boundaries. In this study, design boundaries are investigated using these different fabrication methods and materials. This section summarizes the two fabrication methods.

### Fabrication method of FSPAs via heat bonding

**Figure 1:**
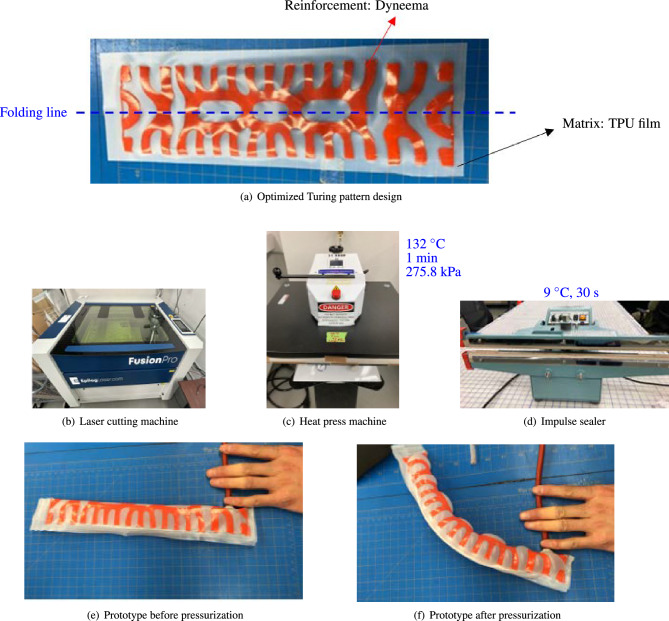
Overview of fabrication method of FSPA via heat bonding: (**a**) Optimized shape of the Turing pattern to achieve bending after pressurization. (**b**) A laser cutting machine that cuts the optimized Turing pattern shape from stiff Dyneema fabric using laser. (**c**) A heat-press machine that bonds two different fabrics, Dyneema and TPU film, by heat pressing. (**d**) A heat sealer that seals the edge lines to form a closed surface through heat sealing. (**e**) Undeformed state of the prototype, equipped with a syringe nozzle. (**f**) Deformed state of prototype, displaying a bending shape after compressed air injection using a pressure dispenser through syringe nozzle.

Here, one of proposed fabrication techniques is illustrated, namely the heat bonding method, as conceptually depicted in Fig. 1. To elaborate, a stiff Turing pattern texture is created on a soft matrix membrane, enabling bending behavior when inflated (Fig. 1a). This Turing pattern is obtained from orientation optimization and the texturing technique, which will be discussed later.

Firstly, two overlapping elastic flat sheets are prepared, one stiff and the other soft, as depicted in Fig. 1a. A stiff fabric Dyneema is laser cut (Fig. 1b) into the optimized Turing pattern shape and placed onto a soft base fabric, Thermoplastic Polyurethane (TPU). The Turing pattern is symmetrically arranged against a folding line, denoted by a blue dotted line in Fig. 1a. The stiff reinforcements, Dyneema, and base soft material, TPU film, are bonded by heat press machine at 132 degrees Celsius and 275.8 KPa for a minute (Fig. 1c). The sheet is then folded along the blue dotted line, aligning the Turing pattern shapes on the upper and lower surfaces due to its symmetrical design. The edges are sealed at 93 degrees Celsius for 30 seconds using a impulse sealer, forming a closed surface (Fig. 1d). Fig. 1e illustrates the completed prototype of the shape-morphing inflatable membrane structure with a one-way valve inserted at one end. The FSPA is then inflated by a Dewalt DCC020IB 20V MAX cordless air inflator. As shown in Fig. 1f, the prototype exhibits the expected bending behavior when internal pressure is applied.

### Fabrication method of FSPAs via embroidery

**Figure 2:**
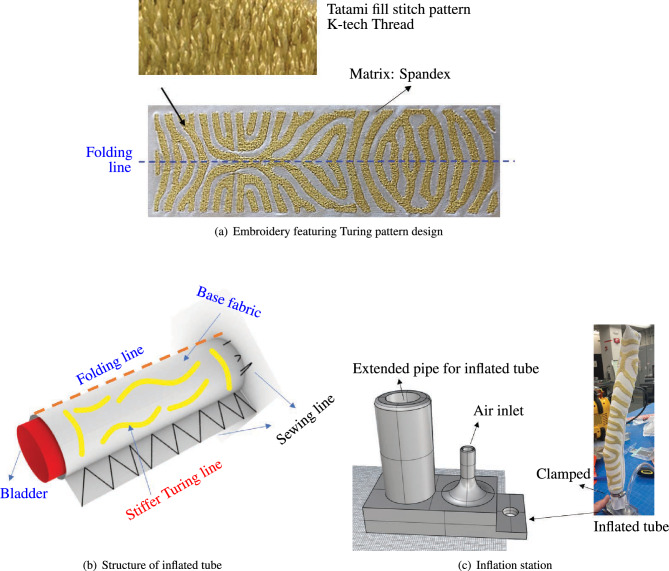
Overview of fabrication method of FSPA via embroidery. (**a**) Embroidery featuring Turing pattern is designed to facilitate bending after pressurization. Tatami fill stitch type is used for this application. (**b**) Structure of inflated tube consists of an embroidered sheet featuring a Turing pattern. (**c**) An inflation station is used to apply air pressure into the inflated tube. The tube is connected to an extended pipe, with an air dispenser attached to its air inlet.

Embroidery and sewing techniques have expanded their applications beyond traditional textile and fashion industries, finding their way into diverse sectors like automotive manufacturing, such as airbags, steering wheels, seat covers or interior decorations. In these applications, specialized sewing machines and embroidery equipment are used to ensure precision, durability, and aesthetic appeal. Moreover, advancements in technology have enabled automated embroidery machines to create intricate designs with high accuracy, meeting the demands of the automotive industry for both functional and aesthetic purposes. Inspired from these applications, the embroidery technique was used for fabrication of the FSPA parts, as shown in Fig. 2.

The study focused on programming embroidery designs. This began with simplifying each design using Adobe Illustrator to ensure compatibility with the embroidery software. The designs were then imported into the software, where stitching patterns were programmed, particularly employing a Tatami fill stitch (Fig. 2a) suitable for larger needles and threads.

Additionally, a ZSK Sprint series machine was specifically adapted for this embroidery work. The execution of the design involved preparing the fabric with three layers of backing/stabilizer and operating the machine at a reduced speed to accommodate the thread’s tendency to shred.

In the final stage, the sheet featuring the embroidery is folded along the folding line, and its edges are stitched with a general sewing machine to create a closed surface. During this process, a bladder is inserted to apply internal pressure as shown in Fig. 2b. The constructed inflatable tube is set up at an inflation station (as shown in Fig. 2c) to introduce air pressure into the tube. This tube is linked to an extended pipe, and an air dispenser is connected to the air inlet.

## Materials

In this section, the materials used in this study for heat-bonding and embroidery fabrications are summarized. This study focuses on the selection of materials to satisfy specific mechanical requirements for both fabrication methods.

Table 1 shows the material information used in the heat-bonding fabrication method. The material used for the matrix is soft fabric Thermoplastic Polyurethane (TPU), with a modulus of elasticity $E_m=10$ MPa and a thickness of 0.3 mm. The material used for reinforcement is stiff fabric Dyneema, with a modulus of elasticity $E_f=8.0$ GPa and a thickness of 0.25 mm.

**Table 1:** Material information used in heat-bonding fabrication method.

	Material name	Young’s modulus	Thickness
Matrix	Thermoplastic Polyurethane (TPU)	10 MPa	0.3 mm
Reinforcement	Dyneema	8 GPa	0.25 mm

Table 2 shows the material information used in the embroidery fabrication method. The primary matrix material was spandex, mainly composed of Polyurethane polymer, which exhibited a Young’s modulus ($E_m$) of approximately 10 MPa. This polymer was selected for its notable elasticity and durability. The thickness of the spandex is 0.2 mm. To complement this, a reinforcement material, Kevlar threaded fabric (K-tech 75 Tex 40 with diameter of filament thread at 12 $\mu$m) known for its high strength and lightweight properties, was used. This reinforcement had a significantly higher Young’s modulus ($E_f$), in the range of about 70 GPa. They are stitched using the Tatami fill pattern.

**Table 2:** Material information used in embroidery fabrication method.

	Material name	Young’s modulus	Thickness/Diameter
Matrix	Spandex	10 MPa	0.2 mm (thickness)
Reinforcement	K-tech thread	70 GPa	0.012 mm (diameter)

## Results

## Validation through three different deformation shapes

**Figure 3:**
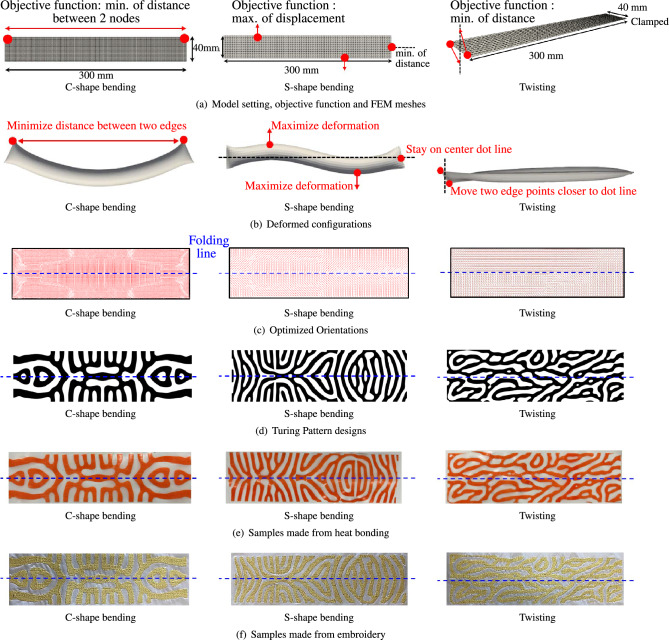
Design and fabrication of proposed method using three types of programmable deformations with Turing patterns: C-shaped and S-shaped bending, and twisting behaviors after inflation. (**a**) Setting of size, objective functions, boundary conditions, and FEM meshes; (**b**) Deformed configurations after orientation optimization; (**c**) Continuous distribution of optimized material orientations; (**d**) Generated Turing patterns; (**e**) Experimental implementation using heat-bonding; (**f**) Experimental implementation using embroidery.

In this section, proposed approach to designing and fabricating pre-programmed inflatable membrane structures is demonstrated and validated, utilizing the control provided by geometric parameters in Turing patterns. Central to proposed methodology is the ability to manipulate the inflated shape of these structures. In this study, the performance of FSPAs with programmable Turing pattern textures is evaluated through three different deformation shapes: C-shaped bending, S-shaped bending, and twisting. The motivations behind targeting these deformation shapes are summarized as follows. The C-shaped bending in the actuators is driven by the need for simple yet effective bending motion that can mimic natural movements, such as bending limbs or flexing structures. This shape is typically useful in applications where a single directional bend is required, such as in robotic arms or supportive braces that adapt to the wearer’s movements. The S-shaped bending is targeted for its complex, double-curved nature, allowing actuators to handle more intricate tasks that require multiple points of articulation within a single component. This deformation is particularly useful in scenarios where nonlinear flexibility and movement are crucial, such as in adaptive piping systems or wearable technology that must conform to the human body’s natural contours. The twisting is aimed at enabling the actuators to perform rotational movements, which are essential in tasks that involve twisting motions. Figure 3 illustrates the design and a part of fabrication process of proposed method for three distinct types of programmable deformations: C- and S-shaped bending, as well as twisting behaviors, each of which becomes observable after inflation.

The flat plane consists of two overlapping sheets: an upper surface and a lower surface, both meshed in the same manner. The nodes on the edge belong to both surfaces. When inflated, the upper and lower surfaces separate outward from each other, forming a closed surface. For C-shaped bending, the objective function is defined as the distance after deformation between two nodes, one on each edge. Minimizing this objective function results in a deformed configuration exhibiting C-shaped bending, as illustrated in Fig. 3b. Similarly, for S-shaped bending, the objective function focuses on the deformation magnitude at three specific nodes (refer to Fig. 3a). Two of these nodes are positioned at one-third the distance from each end, and the third node is located at the central point of one end edge. The objective is to maximize outward deformations at the first two nodes while minimizing the deformation at the last node. Achieving these objectives results in the deformed configuration assuming an S-shape, as depicted in Fig. 3b. Regarding twisting, the objective function is to maximize the out-of-plane deformations at two edge nodes on one end, while the other end is clamped (Fig. 3a). This involves upward deformation at one node and downward deformation at the other. Additionally, the distance of these two nodes in the deformed configuration to the center line should be minimized. Achieving this objective results in a deformed configuration displaying twisting behavior, as illustrated in Fig. 3b.

Orientation optimization was conducted to achieve these objectives. Figure 3c illustrates the optimization outcomes for the C-shaped and S-shaped bending, as well as the twisting morphing structures. The nonlinear shell FEM in the orientation optimization loop is implemented using in-house code. In this process, continuous orientation fields that guide the formation of the inflated structure are derived. It is important to note that for C- and S-shaped bending, the distribution of optimized orientations on the upper and lower surfaces is identical, reflecting the symmetric nature of the deformed shapes against these surfaces. However, in the case of twisting, the distribution on the upper surface is distinct from that on the lower one due to the asymmetric nature of the deformation.

Figure 3d presents the discretized Turing patterns corresponding to the material orientations for C-shaped and S-shaped bending, as well as twisting, derived from anisotropic reaction-diffusion equations. These anisotropic reaction-diffusion equations are solved using COMSOL Multiphysics. These patterns play a vital role as they determine the final shape and behavior of the inflated structure. The black areas represent stiff material, while the white areas indicate softer material. To aid in practical implementation, Computer Aided Design (CAD) data for these Turing patterns is generated.

The prototypes of these structures were fabricated from the aforementioned CAD data using two methods: heat bonding and embroidery techniques, as illustrated in Fig. 3e and f. Figure 3e depicts prototypes produced through the heat bonding technique. In this method, Dyneema, a relatively stiff fabric, is laser-cut into the Turing pattern geometry and then adhered to a TPU film base fabric, a softer material, using a heat press. In contrast, Fig. 3f demonstrates prototypes crafted using the embroidery technique, where the black areas of the Turing pattern are stitched with stiff thread (K-tech 75 Tex 40) onto soft base fabrics (Polyurethane polymer).

The final step in the fabrication process for both methods involves sealing the edges with a heat-sealer, following the installation of a valve for air inflow.

**Figure 4:**
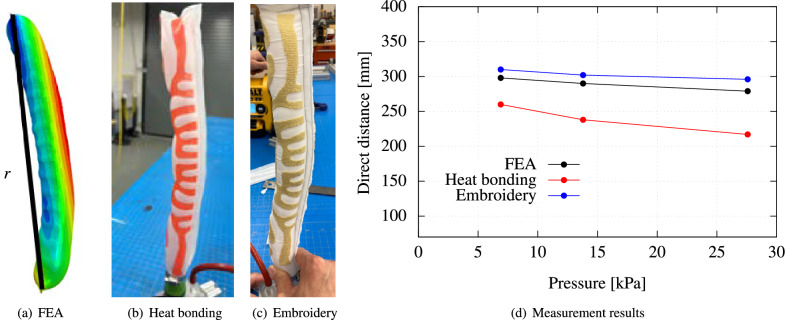
Experimental validation of a programmable FSPA achieving C-shaped bending after inflation: (**a**) Finite element analysis (FEA) simulation; (**b**) Experimental observation of deformed prototype fabricated using heat-bonding technique and (**c**) using embroidery technique; (**d**) Comparison of direct distance *r* between two edges and internal pressure relationships.

**Figure 5:**
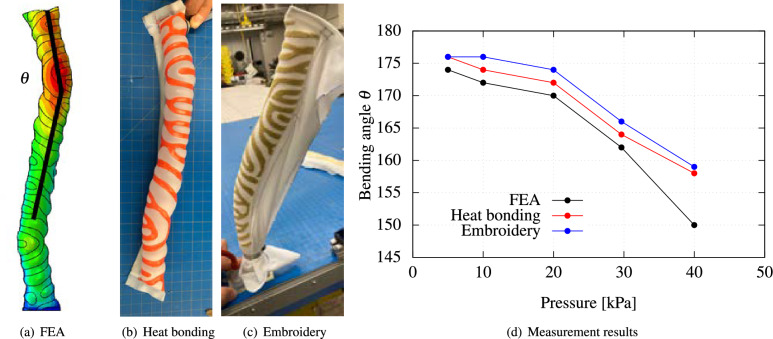
Experimental validation of a programmable FSPA achieving S-shaped bending after inflation: (**a**) FEA simulation; (**b**) Experimental observation of deformed prototype fabricated using heat-bonding technique; (**c**) using embroidery technique; (**d**) Comparison of the bending angle $\theta$ at the first inflection point and internal pressure relationships.

**Figure 6:**
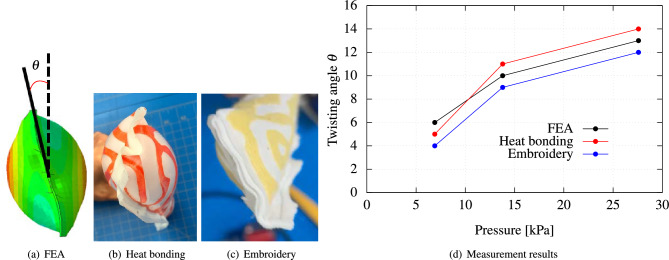
Experimental validation of a programmable FSPA achieving twisting bending after inflation: (**a**) FEA simulation; (**b**) Experimental observation of deformed prototype fabricated using heat-bonding technique; (**c**) using embroidery technique; (**d**) Comparison of twisting angle $\theta$ and internal pressure relationships.

The experimental validation of the deformed configurations of these structures demonstrates the effectiveness of present approach. Figs. 4, 5, and 6 illustrate the C-shaped bending, S-shaped bending, and twisting behaviors, respectively, of these prototypes upon inflation. In these figures, detailed FEM simulations using Abaqus are also shown for comparison.

Figure 4 illustrates the results concerning the C-shaped bending morphing structure. This includes FEM simulation of the structure, experimental observations of the deformed prototypes fabricated using both heat-bonding and embroidery techniques, and a comparison of the direct distance between two edges and internal pressure is conducted for the simulations and the actual prototypes. In Fig. 4a, the FEM model is illustrated with a Turing pattern mesh applied to the membrane. The direct distance between two edges is computed and displayed in Fig. 4d. These calculations are then juxtaposed with direct distances measured from experimental prototypes, which were analyzed using image processing at identical simulation points, as depicted in Fig. 4b and c. The collected data is graphically represented, showing a close correlation between direct distances and internal pressures as highlighted in Fig. 4d.

Figure 5 shifts focus to the S-shaped bending structure. Employing similar methods, it details the FEM simulation and empirical measurements from prototypes, as shown in Fig. 5a–c. The primary bending angle, as elaborated in Fig. 5d, underlines the alignment between the simulation and physical data, confirming the reliability of the bending pattern observations.

The comparison then moves to the twisting morphing structure, captured in Fig. 6. The twisting angles, observed from the FEM model’s top view (Fig. 6a), are juxtaposed with those from experimental prototypes (Fig. 6b and c). The consistency between these two sets of data is underscored in Fig. 6d, emphasizing the influence of internal pressure on twisting angles.

In summary, the application of Turing patterns in these morphing structures demonstrates their utility in fabricating programmable shapes. This approach’s potential is vast, with promising applications across diverse domains, marking a significant step forward in inflatable structure design.

## Comparison with classical simple designs

**Figure 7:**
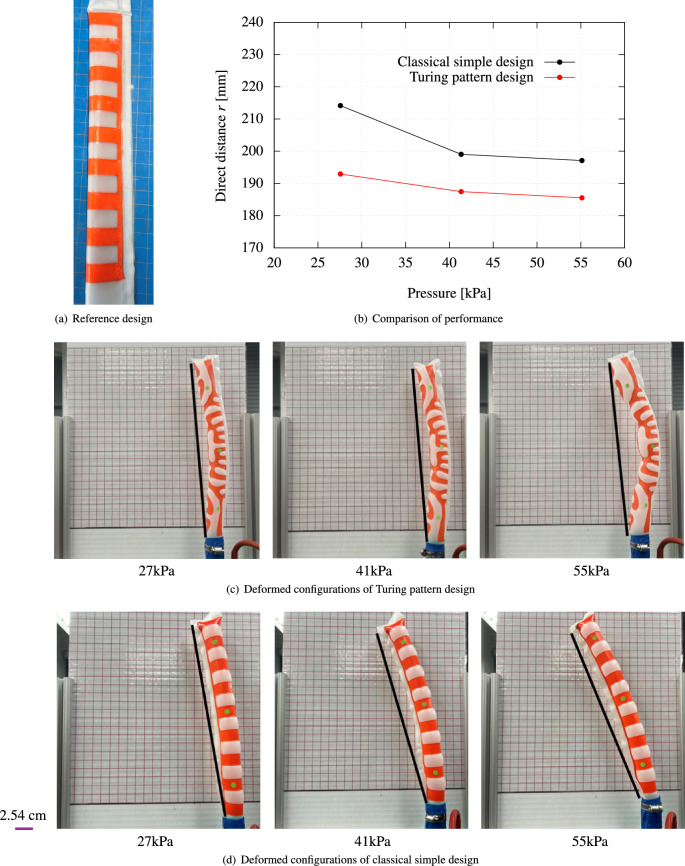
Comparison of performance between reference design^1^ and proposed Turing pattern designs for C-shaped bending. (**a**) Reference design: Layout of reference design features a checkered pattern with reinforcement strips. (**b**) Comparison of performance between reference model and proposed Turing pattern design model. (**c**) Deformed configurations of proposed Turing pattern design. (**d**) Deformed configurations of classical simple reference design.

**Figure 8:**
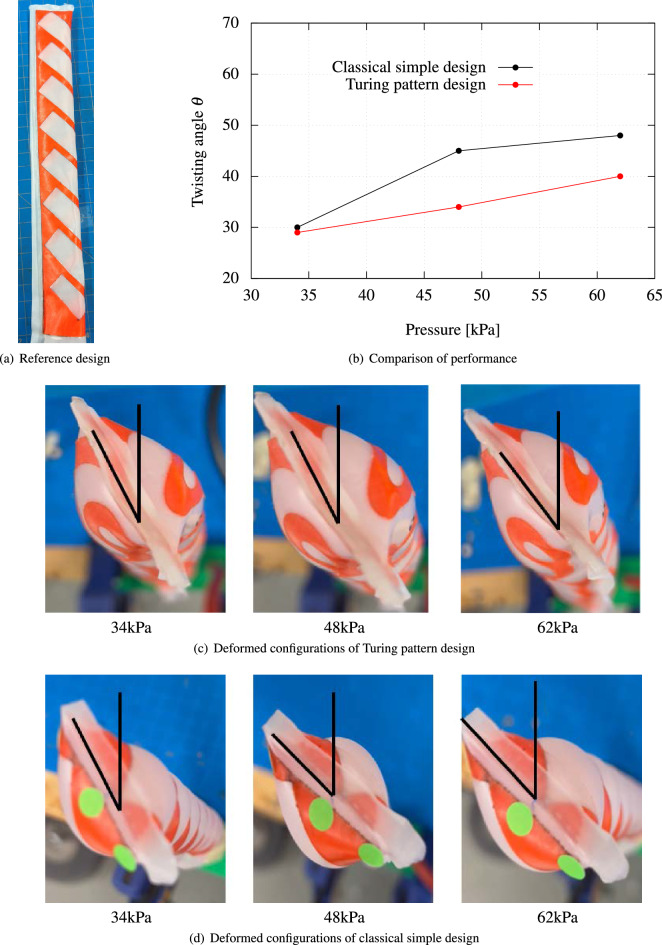
Comparison of performance between reference design^1^ and propsed Turing pattern designs for twisting. (**a**) Reference design: layout of reference design features a diagonal checkered pattern with reinforcement strips. (**b**) Comparison of performance between reference model and proposed Turing pattern design model. (**c**) Deformed configurations of proposed Turing pattern design. (**d**) Deformed configurations of classical simple reference design.

In this section, to assess the optimized performance of the proposed Turing pattern designs, classical and simple designs found in the literature are compared with these patterns, which also employ only air input and do not require specialized materials. The design model from the literature^1^ uses a simple stripe design and is considered the best fit in this context. Figures 7a and 8a illustrate manual designs for C-shaped and twisting deformations as per previous research^1^. For the C-shaped bending, the horizontal stripe design is employed, and for the twisting, the diagonal stripe design. The materials used in these designs are exactly the same in order to purely compare performance based on the designs themselves.

Figures 7b and 8b compare the performance of the present method with previous research for both C-shaped bending and twisting. For the C-shaped bending, note that the objective function is the direct distance between two edges, and therefore the prototype with the present Turing design showed better performance in terms of the minimization of the direct distance, which can be clearly confirmed by photographs in the measurements in Fig. 7c and d. It can be said that the Turing pattern design showed better optimized performance than the classical and simple design.

In the case of twisting, the prototype with the classical design showed a bit more twisting angle with the internal pressure of 41 and 55 kPa. However, as shown in Fig. 8c and d, the twisting behaviors of both prototypes are almost identical to the naked eye, and thus it can be said both designs have similar performance, and the difference might be due to experimental and fabrication errors.

In summary, the present method with the Turing pattern design showed better or similar optimized performance compared to classical and simple designs. Furthermore, it is worth noting that the present method can derive counterintuitive inflated shapes such as the S-shaped bending with the proposed optimization procedure.

## Summary and discussion

This study introduces a method for programming and manufacturing fabric-based inflatable, shape-morphing membrane structures. By fine-tuning the geometric pattern of a reinforcement sheet embedded into an unstructured elastomeric membrane, the local deformations of the surface membrane can be controlled, thereby dictating its overall shape.

Initially, an optimized distribution of the material orientation field on the surface membrane is achieved through gradient-based orientation optimization. This involves a comprehensive physical deployment simulation using the nonlinear shell finite element method, which is integrated into the inner loop of the optimization algorithm. This continuous adjustment of material orientations enhances the design objectives.

However, challenges arise with the direct 3D printing of anisotropic materials, which control local material orientation. Special post-processing may be required to ensure continuity while utilizing 3D printing from the optimized distribution field of material orientation.

To address this, Turing pattern textures are employed to print anisotropic material fields on inflatable structure surfaces. These material orientation fields are transformed into discretized texture patterns that replicate the same anisotropic deformations. Anisotropic reaction-diffusion equations, using diffusion coefficients determined by local orientations from the optimization step, are then utilized to create space-filling Turing pattern textures. This technique offers a solution for the 3D printing of anisotropic FSPAs that directly generates fabrication instructions.

In this study, two different fabrication methods are explored : heat bonding and embroidery. The heat bonding method, using Dyneema for its high modulus and TPU for bonding strength and airtightness, presents challenges in finding two contrasting-stiffness fabric materials that can be heat-pressed together effectively. For the embroidery method, Kevlar fiber was selected as the optimal material considering the constraints such as needle size, stiffness, and compatibility with the base fabric.

Proposed designs, derived from a gradient-based material orientation optimization method, represent local rather than global optima. The generation of the binarized Turing pattern, achieved by tuning parameters in reaction-diffusion equations, considers the need for lower resolutions in fabric implementations. These patterns approximate the optimized material orientation distributions, facilitating easier implementation and lighter weight. As a result, while the “best” performance cannot be guaranteed, focus remains on ease of implementation, lightness, and aesthetic appeal. Furthermore, the proposed design method can automatically generate less obvious designs, such as S-shaped bending.

Some bio-inspired muscular-type manipulators, such as octopus arms or elephant trunks, have been discussed in both academic literature^40–42^ and commercial products^43^. These examples demonstrate significant deformations and precise motions. However, they utilize specialized materials and technologies such as shape memory polymers. In contrast, present simpler approach, relying solely on air input and surface texture patterns on membrane fabrics, avoids specialized materials, leading to cost-effective implementations.

## Conclusion

This study introduces a novel approach to the design and fabrication of FSPAs using programmable Turing patterns. The innovative methods developed leverage the morphogenetic principles first proposed by Alan Turing, applying them to create complex, controlled movement patterns in soft robotic structures using only membrane surface textural patterns, without the need for specialized materials.

Current research highlights the underutilization of Turing patterns in industrial applications, despite their potential to enhance the functionality and adaptability of textile technologies. By integrating these patterns into the fabric of soft actuators, the ability to pre-program dynamic movements and achieve precise control over material behavior at the micro-level is demonstrated. This capability represents a substantial advancement over traditional textile actuator technologies, which are often constrained by the limitations of their design and manufacturing processes.

The methods developed in this study-specifically, the optimization of material orientation and the innovative use of heat bonding and embroidery in fabrication-significantly enhance the design and manufacturing processes. These techniques not only meet but also exceed the design objectives by enabling FSPAs to perform complex deformations such as C- and S-shaped bending and twisting more reliably.

Furthermore, the application of advanced numerical optimization techniques for the automatic design of these structures opens new avenues for research and development in soft robotics. The outcomes of this study provide a foundation for future innovations in the field and highlight the potential for Turing patterns to revolutionize the design and functionality of soft robotic systems.

This research not only fills a crucial research gap but also sets a new benchmark for what can be achieved in the domain of soft robotics, pushing the boundaries of traditional design and manufacturing techniques to accommodate the growing demands of next-generation robotic systems.

In the outlook, different drive forces besides air inflation will be investigated with current Turing pattern designs using modern material technologies such as shape memory functions.

## Data Availability

The datasets used and/or analysed during the current study available from the corresponding author on reasonable request.
